# Comparative Study on Beneficial Effects of Hydroxytyrosol- and Oleuropein-Rich Olive Leaf Extracts on High-Fat Diet-Induced Lipid Metabolism Disturbance and Liver Injury in Rats

**DOI:** 10.1155/2020/1315202

**Published:** 2020-01-08

**Authors:** Ines Fki, Sami Sayadi, Asma Mahmoudi, Ines Daoued, Rim Marrekchi, Hela Ghorbel

**Affiliations:** ^1^Laboratory of Environmental Bioprocesses, Center of Biotechnology of Sfax, University of Sfax, P.O. Box 1177, 3038 Sfax, Tunisia; ^2^Center of Sustainable Development, College of Arts and Sciences, Qatar University, Doha 2713, Qatar; ^3^Laboratory of Biochemistry, Chu Hédi Chaker, 3029 Sfax, Tunisia

## Abstract

Oleuropein and hydroxytyrosol, as major compounds of olive leaves, have been reported to exert numerous pharmacological properties, including anticancer, antidiabetic, and anti-inflammatory activities. The purpose of this study is to evaluate and compare the protective effect of oleuropein- and hydroxytyrosol-rich extracts, derived from olive leaves, on high-fat diet-induced lipid metabolism disturbance and liver injury in rats. In this respect, four groups of male rats (8 per group) were used: control group (Control), group treated with high-fat diet (HFD), group treated with HFD and oleuropein (HFD + OLE), and group treated with HFD and hydroxytyrosol (HFD + HYD). The current research showed that the treatment with the HFD increased the body weight and adipose tissue mass in male rats. Moreover, the plasma levels of triglycerides, total cholesterol, LDL-cholesterol, AST, ALT, LDH, and TNF-*α* were also raised. The hepatic immunohistochemical analysis revealed a significant increase in the expression of inflammatory genes (COX-2, NF-*κ*B, and TNF-*α*). Equally, it showed a rise of the apoptotic markers (a decrease in the expression of the Bcl-2 and an increase of the P53). In addition, the oral administration of oleuropein- and hydroxytyrosol-rich olive leaf extracts at 16 mg/kg similarly reduced the body weight and adipose tissue mass and improved the lipid profile. Moreover, these extracts, mainly the hydroxytyrosol-rich extract, reduced the elevated liver enzymes, enhanced the antioxidant status, and attenuated the liver inflammation and apoptosis. These findings suggest that the oleuropein- and hydroxytyrosol-rich olive leaf extracts possessed hypolipidemic and hepatoprotective effects against the HFD-induced metabolic disorders by enhancing the antioxidative defense system and blocking the expression of the proteins involved in inflammation and liver damage.

## 1. Introduction

Obesity is a serious health problem worldwide that increases the risk factors for chronic diseases such as diabetes, cardiovascular disease, liver dysfunction, hypertension, and some forms of cancer [1–4]. Even though the pathophysiology of obesity is complex, dietary factors, mainly the consumption of high-fat diet, are considered as major risk factors for its development. Consequently, it is very important to prevent obesity for a healthy life. Many different approaches have been used to treat obesity, including diet control, sport, behavior therapy, medications, and surgery [5–7]. Among these therapies, change in eating habits, exercise, and behavior modification are essential to the management of obesity. Nevertheless, their outcomes are not often satisfactory. Various antiobesity drugs, including orlistat, phentermine, mazindol, phendimetrazine, and diethylpropion, have been approved by the Food and Drug Administration. However, since these drugs have been known for their side effects such as headaches, vomiting, and myocardial infarctions. Orlistat is the only one approved due to its long-term weight control [8]. Numerous researchers are interested in using effective natural products based on fruits, vegetables, and herbs for the prevention and/or treatment of obesity.

In the Mediterranean area, olive leaves are one of the by-products of the olive grove farming. They can be found in high amounts in the olive oil industries (10% of the total weight of the olives) and they are collected during the pruning of the olive trees [9]. The olive leaf has been commonly used in folk remedies as well as in human diet as an extract, in herbal tea and in the powder form in the Mediterranean countries [10]. Current studies have proved that olive leaves are considered as an economical raw material which can be regarded as a useful source for high added value products [11].

Olive leaf extracts have drawn the attention of a number of researchers. It is mainly due to the typical phenolic composition linked to potent biological activities [12]. The major active component in olive leaves is oleuropein. After the hydrolysis, oleuropein can produce other bioactive substances, especially hydroxytyrosol [13]. Numerous *in vitro* and *in vivo* studies have revealed that oleuropein and its derivative hydroxytyrosol possess a wide range of biochemical and pharmacological properties. In fact, oleuropein and hydroxytyrosol have previously been reported to have antiproliferative, anti-inflammatory, antidiabetic, and hypocholesterolemic properties [14–16].

Furthermore, several in vivo studies have demonstrated the protective effect of hydroxytyrosol and oleuropein on body weight gain and metabolic impairment in a rat model of obesity [17, 18]. However, there are no researches focusing on the comparison of the preventive role of hydroxytyrosol and oleuropein on the development of obesity and the associated metabolic complications.

Therefore, the aim of this study was to compare the protective effect of the oleuropein- and hydroxytyrosol-rich extracts derived from olive leaves on the high-fat diet-induced lipid metabolism disturbance and liver injury in rats.

## 2. Materials and Methods

### 2.1. Oleuropein-Rich Olive Leaf Extract Preparation

*Olea europaea* leaves from *chemlali* cultivar were collected from the area of Sfax (Southeast of Tunisia). Olive leaves were dried under microwaves and powdered for extraction. 100 g of ground olive leaves were immersed in 500 mL distilled water. The mixture was stirred at room temperature overnight. Then it was filtered and the aqueous phase was extracted thrice with an equal volume of ethyl acetate. After drying the organic phase under vacuum, the residue was lyophilized and stored until use.

### 2.2. Hydroxytyrosol-Rich Olive Leaf Extract Preparation

The olive leaves powder was extracted with a mixture of methanol and water (4 : 1, vol/vol) overnight under agitation. Next to that, the filtrate was hydrolyzed at 100°C for 1 h by using 2 M HCl (4 : 1 vol/vol). The mixture was cooled and then extracted three times with the ethyl acetate, which was removed by evaporation. The residue was stored for further analyses.

### 2.3. HPLC Analysis

A high performance liquid chromatography analysis was performed in order to recognize and quantify the major phenolic compounds of the olive leaf extract. The phenolic profile was taken following the method of Souilem et al. [19] by using an Agilent series 1260 HPLC-DAD instrument (Agilent Technologies, Waldbronn, Germany). The mobile phase was made of both phase A (0.1% acetic acid in water) and phase B (100% acetonitrile). The elution conditions were as follows: the flow rate set at 0.5 ml/min, injection volume of 10 ml, and operating temperature of 40°C. The running gradient was as follows: 0–22 min, 10–50% B; 22–32 min, 50–100% B; 32–40 min, 100% B; 40–44 min, 100–10% B.

Detection was conducted by a diode array detector (DAD) while the chromatograms were recorded at *λ* = 280 nm for oleuropein and hydroxytyrosol and at 330 nm for flavonoids. The act of quantification was carried out by external calibration with particulars standards. The purity level of oleuropein and hydroxytyrosol was determined using the LCMS/MS (Agilent 1100 LC, Germany) equipment according to Bouallagui et al. [14].

### 2.4. Animals and Treatments

Male 10-week-old Swiss rats weighing about 240 ± 10 g were obtained from the Central Pharmacy (SIPHAT, Tunisia). All animal procedures were conducted in accordance with the European Convention for the protection of vertebrate animals used for experimental and other scientific purposes (Council of Europe no. 123, Strasbourg, 1985). The approval of these experiments was obtained from the Medical Ethics Committee for the Care and Use of Laboratory Animals of the Pasteur Institute of Tunis, Tunisia (approval number: FST/LNFP/Pro 152012). They had free access to food and water for two weeks to acclimatize them to the new environment. The rats were randomly divided into four groups of eight. The first group served as the control (CD), received normal water, standard diet supplied by the Company of Animal Nutrition, Sfax, Tunisia. The second group received a high-fat diet (HFD) (normal diet supplemented with 17% of sheep fat, 3% corn oil, and 0.1% bile salts) (Table 1). The third group received a high-fat diet and treated with the oleuropein-rich olive leaf extract (The quantity succeeding to 16 mg/kg of B.W. oleuropein). The fourth group received a high-fat diet and treated with the hydroxytyrosol-rich olive leaf extract (the quantity succeeding to 16 mg/kg of B.W. hydroxytyrosol). The treatment with the oleuropein- and hydroxytyrosol-rich olive leaf extracts was via oral route for 60 days.

### 2.5. Samples Preparation

During the experiment, the body growth of rats was continuously monitored. After 60 days of treatment, blood samples were collected from the brachial artery in heparinized tubes and centrifuged at 2200*g* for 15 min. The liver and epididymal adipose tissue were carefully dissected out and weighed. All the samples were stored at −80°C for subsequent biochemical and histological analyses.

### 2.6. Biochemical Analysis

The plasma levels of the aspartate aminotransferase (AST), lactate dehydrogenase (LDH), alanine aminotransferase (ALT), triglyceride (TG), total cholesterol (TC), HDL-cholesterol, and glucose were measured by using an automatic biochemistry analyzer (Vita lab Flexor E, USA) at the biochemical laboratory of the Hedi Chaker Hospital (Sfax, Tunisia). The plasma levels of the TNF-*α*, leptin, and insulin were determined by the immunoassays as well as by using a commercially available ELISA kit from BioVendor Research and Diagnostic Product. The insulin resistance was estimated by means of the Homeostasis Model Assessment Index for Insulin Resistance (HOMA-IR). It was done so by the following formula as described by Cacho et al. [20]:

(1) $$\begin{array}{c} \text{HOMA IR index}=\frac{\left[\text{fasting glucose}\left(\text{mmol}/\text{L}\right)\times \text{fasting insulin}\left(\text{mU}/\text{ml}\right)\right]}{22.5}. \end{array}$$

### 2.7. Liver Cytosol Extraction

10 g of the liver sample was homogenized in cold KCl (1.15%, pH: 7.4) supplemented with the protease inhibitor cocktail (Sigma-Aldrich) by using Polytron (PT2500E) homogenizer. The homogenates were centrifuged at 14000*g* and 4°C for 45 min. The supernatants were preserved at −80°C for further analyses.

### 2.8. Determination of Liver TBARS

As a marker of lipid peroxidation, the TBARS (thiobarbituric acid-reactive substances) concentrations were measured in the liver cytosol. Briefly, 200 *μ*l of cytosolic samples was blended with 600 *μ*l of distilled water and 200 *μ*l of 8.1% (w/v) SDS, vortexed, and then incubated at room temperature for 5 min. The mix was heated at 95°C for 1 h after the addition of 1.5 ml of 20% acetic acid (pH: 3.5) and 1.5 ml of 0.8% (w/v) thiobarbituric acid (TBA). After incubation, the reaction was cooled and 1 ml of distilled water and 5 ml of butanol: pyridine (15 : 1) solution were supplemented. The mixture was centrifuged at 1935*g* for 15 min and the obtained colored layer was measured at 532 nm by using the malondialdehyde (MDA) made by the hydrolysis of 1,1,3,3-tetramethoxypropane as standard.

### 2.9. Total Antioxidant Capacity of Liver

The Trolox equivalent antioxidant capacity (TEAC) assay measured the reduction of the ABTS radical cation by antioxidants. ABTS radical cation (ABTS+) was formed by reacting 7 mM ABTS stock solution with 140 mM potassium persulfate, allowing the mixture to stand in the dark at a room temperature for 12–16 h before use. For the current study, the ABTS + solution was diluted with ethanol to an absorbance of 0.70 (±0.02) at 734 nm. In the reaction, 1 ml of diluted ABTS + solution was supplemented to 50 *μ*l cytosol samples or Trolox standard. The mixture was incubated for 2 min in a glass cuvette at 30°C. The decrease in absorbance was recorded at 734 nm. All the measurements were executed in triplicate. The free radical scavenging capacity of the biological samples, calculated as inhibition percentage of ABTS+, was determined against a Trolox standard curve prepared with different concentrations (40–200 *μ*mol/l). The results were expressed as *μ*M of Trolox equivalents.

### 2.10. Assay of Antioxidant System

Catalase (CAT) activity was evaluated according to Aebi [21]. Briefly, 20 mL of liver homogenates was added to 1 mL of 100 mM H_2_O_2_ in 0.1 M phosphate buffer (pH 7.0). The H_2_O_2_ decomposition rate was followed by measurement of the decline in absorbance at 240 nm for 1 min. CAT activity was expressed in international units (IU), that is, in mmol H_2_O_2_/min/mg protein.

Superoxide dismutase activity (SOD) was calculated by the method of Beauchamp and Fridovich [22]. Superoxide radicals react with nitro blue tetrazolium (NBT) in the presence of nicotinamide adenine dinucleotide hydrate (NADH) and they produce formazan blue. The SOD eliminates the superoxide radicals and inhibits the formation of formazan blue. The intensity of the color is inversely proportional to the enzyme activity. It is read at 560 nm. One unit of enzyme activity was taken as the enzyme reaction, which gave 50% inhibition of NBT reduction in one min in the assay conditions. It is expressed as specific activity in units/mg protein.

### 2.11. Histopathological Examination

The liver and epididymal adipose tissues from all groups were fixed in 10% formalin liquid, and embedded in paraffin. Embedded tissues were cut by using microtome at 4 *μ*m thickness and next mounted on slides. All sections were deparaffinized and rehydrated by submerging the slides in decreasing concentrations of ethanol. Subsequently, the slides were examined through hematoxylin-eosin (H&E) staining. Digital images were obtained with an Olympus BX 51TF microscope (Olympus Corporation, Tokyo, Japan).

The volume densities of liver steatosis (Vv[steatosis, liver]) were assessed by point counting methods as mentioned previously [23]. Briefly, the volume densities of hepatic steatosis were estimated with a test system made up of 36 test points (PT) produced by the STEPanizer web-based system [24]. The following equation was employed:

(2) $$\begin{array}{c} \text{Vv}\left[\text{steatosis,liver}\right]=\frac{\text{Pp}\left[\text{steatosis}\right]}{\text{PT}}, \end{array}$$

Pp is the number of points that hit the structure, and PT is the total test points.

The average cross-sectional area of the adipocytes was evaluated by stereology as the ratio between the volume density of adipocytes (Vv[adipocyte]) and twice the numerical density per area of adipocytes (QA[adipocyte]). Vv[adipocyte] was estimated by point counting on a test system, and QA[adipocyte] was estimated as the ratio between the number of adipocytes counted into a frame (without hitting the “forbidden line”) and the test area of the frame [25].

The numerical density and mean nuclear height of adipocytes were assessed by stereological analyses as mentioned previously [26]. Briefly, physical dissector pairs were selected and pairs from every fifth section were chosen randomly. In this way approximately 15–20 section pairs were obtained and evaluated. The dissector pairs were taken from the tissue at a known interval, until the tissue sample was exhausted. Two consecutive sections were mounted on each slide. Photographs of adjacent sections were taken with a digital camera at a magnification of ×400. Nuclei of adipocytes seen in the reference section but not in the look-up section were counted. To increase the countable particle (adipocyte cell nucleus) number, we exchanged the role of sections in the second step. An unbiased counting frame was placed on the reference and the look-up sections on the screen of the PC to perform the counting according to the dissector counting method. The bottom and the left hand edges of the counting frame are considered to be the forbidden (exclusion) lines together with the extension lines. Other boundaries of the frame and the top-right corner were considered to be inclusion points and any particle that hit these lines or was located inside the frame was counted as a dissector particle.

The appropriate size of unbiased counting frame was adjusted to count approximately 600 islet cells from each sample. The dimension of counting frame at PC screen used in this study was 5 cm × 5 cm and the real dimension of this counting frame (15.625 × 10^−5 ^cm^2^) was estimated by the following formula:

(3) $$\begin{array}{c} \text{Real dimension}=\frac{\text{Screen Size of Frame}}{\text{Total Magnification of Microscope}}\text{.} \end{array}$$

The mean numerical density of adipocytes [Nv(adipocyte)] per mm^3^ was estimated using the following formula:

(4) $$\begin{array}{c} \text{Nv}\left(\text{adipocyte}\right)=\frac{\Sigma Q-\left(\text{adipocyte}\right)}{t\cdot A}, \end{array}$$

where Σ*Q*−adipocyte is the total number of nuclei counted in the reference section; *t* is the mean section thickness (1 *μ*m), and *A* is the area of the unbiased counting frame.

The mean nuclear height (*H*(nucleus)), which is a measure of the size of nucleus that depends on the section plane, was estimated by the following equation:

(5) $$\begin{array}{c} H\left(\text{nucleus}\right)=\left[\frac{\Sigma Q\left(\text{nucleus}\right)}{\Sigma Q-\left(\text{nucleus}\right)}\right]\times t, \end{array}$$

where Σ*Q*(nucleus) is the total number of nuclei counted in the reference section, Σ*Q* − (nucleus) is the total number of dissector nuclei counted in the reference section, and *t* is the mean section thickness (1 *μ*m).

### 2.12. Immunohistochemistry

Liver tissues were dewaxed by standard techniques. The heat treatment to recover the antigen sites was carried out. In order to quench endogenous peroxidases, the sections were treated with 3% hydrogen peroxide at room temperature. Next, the sections were incubated for 1 h at room temperature with a blocking solution and next with primary antibodies overnight at 4°C. The primary antibodies incorporated rabbit polyclonal antibody against P53, Bcl-2, COX-2, NF-*κ*B and TNF-*α*. The reactivity of the antibodies was detected with a streptavidin-peroxidase histostaining-SP kit. Positive immunohistochemistry (IHC) stains were defined as yellow-brown color. The IHC staining slides were read on an Olympus optical microscope over yellow-brown color stains for 12 consecutive fields. They were scored according to two variable factors: (1) counting the number of positively stained cells (0 = <5%; 1 = 6%–25%; 2 = 26%–50%; 3 = 51%–75%; and 4 = 76%–100%); and (2) scoring the intensity of the staining (0 = absent; 1 = weak; 2 = moderate; 3 = strong). The final score was the product of (1) multiplying (2) for individual slides according to Li et al. [27]. The final staining scores were expressed in the following ways for simplicity: negative staining as 0 for (−); incremental positive staining as 1 + for (+); 2 + for (++); and 3 + for (+++), respectively [27].

### 2.13. Statistical Analysis

All values were presented as means ± standard deviation (mean ± SD). The data were statistically analyzed by one-way ANOVA, followed by Holm-Sidak post hoc test and by applying a significance level of *p* < 0.05.

## 3. Results

### 3.1. Oleuropein- and Hydroxytyrosol-Rich Olive Leaf Extract Characterization

The phenolic composition of olive leaf extracts was investigated by the HPLC. Peaks were identified by the comparison of the chromatographic retention time and by the UV absorbance spectra of the compounds in olive extracts with those of authentic standards. Tables 2 and 3 listed each of the identified phenolics in an eluted order. These tables illustrated that oleuropein was the main compound detected in the olive leaf extract (OLE). It represented 96.95% of the total phenols. Its concentration attained 905.96 mg/g of OLE. Nevertheless, hydroxytyrosol was the major phenolic constituent of hydrolysate olive leaf extract (HOLE). It represented 87.36% of the total phenols. Its concentration reached 53.29 mg/g of HOLE. Other phenolic compounds such as apigenin-7-O-glucoside and luteolin-7-O-glucoside were detected at lower concentrations in both extracts.

### 3.2. Body Growth and Epididymal Fat Weight

During the treatment period, a regular increase in body growth rate was observed in the HFD treated rats as compared to control group. In fact, a significant increase is noticed in the final body weight and in the body mass index of the HFD treated rats, respectively, by 25.43 and 50%, comparatively to the control group. The oral administration of the oleuropein- and hydroxytyrosol-rich extracts reduced the body weight gain and the body mass index of HFD-OLE and HFD-HYT groups by (63.31%; 16.66%) and (63.60%; 16.66%), respectively, as compared to the HFD treated group (Table 4). To examine the effect of the olive leaf extracts supplement on body fat distribution, the weights of the epididymal fat were measured. The relative weight of epididymal adipose tissue was significantly larger in the HFD group than in the CD group. Supplementation of the HFD rats with oleuropein and hydroxytyrosol significantly decreased their epididymal adipose tissue weight when compared with the high-fat-fed group.

### 3.3. Lipid Parameters

The results of lipid parameters analysis are shown in Table 5. In fact, a significant increase is found in cholesterol, triglyceride, and LDL-cholesterol levels by 53, 169, and 146%, respectively, in the HFD-fed rats as compared to the control group. However, the administration of the oleuropein- and hydroxytyrosol-rich olive leaf extracts led to a significant decrease of the plasma lipid content in comparison to the values found in the high-fat diet fed rats (*p* < 0.001). Comparing the hydroxytyrosol extract to the oleuropein one, it was found more effective in the restoration of lipids levels.

### 3.4. Blood Glucose, Insulin, and HOMA-IR Levels

**Figure 1:**
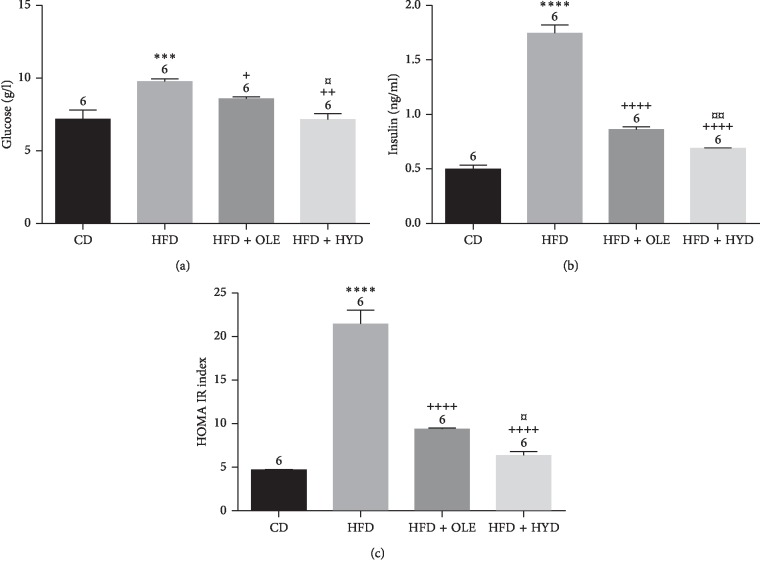
Effect of HFD and oleuropein- and hydroxytyrosol-rich olive leaf extracts on plasma glucose, insulin, and HOMA-IR levels: control group (CD), treated group with HFD (HFD), treated group with HFD and oleuropein (HFD + OLE), and treated group with HFD and hydroxytyrosol (HFD + HYD). Treated (HFD) vs. Control (CD): ^*∗∗∗∗*^*p* ≤ 0.0001, ^*∗∗∗*^*p* ≤ 0.001, ^*∗∗*^*p* ≤ 0.01, ^*∗*^*p* ≤ 0.05. Treated (HFD + OLE, HFD + HYD) vs. Treated (HFD): ^++++^*p* ≤ 0.0001, ^+++^*p* ≤ 0.001, ^++^*p* ≤ 0.01, ^+^*p* ≤ 0.05. Treated HFD + OLE vs. Treated HFD + HYD: ^¤¤¤¤^*p* ≤ 0.0001, ^¤¤¤^*p* ≤ 0.001, ^¤¤^*p* ≤ 0.01, ^¤^*p* ≤ 0.05. (a) Glucose. (b) Insulin. (c) HOMA-IR.

The effects of the olive leaf extracts on the plasma glucose, the insulin, and the HOMA-IR levels of rats in different groups are shown in Figure 1. At the end of the experiment, the HFD-fed rats exhibited a significant increase of glucose, insulin, and HOMA-IR levels as compared to CD group. However, the oleuropein and hydroxytyrosol groups showed lower (*p* < 0.001) plasma levels of glucose, insulin, and HOMA-IR than the HFD group. In comparison to the oleuropein extract, the hydroxytyrosol one was more effective in the restoration of glucose, insulin, and HOMA-IR amounts.

### 3.5. Plasma Levels of Liver Injury Biomarkers

As shown in Table 6, the data illustrated a significant increase in the plasma of AST, ALT, and the LDH levels of the HFD-fed rats (*p* < 0.01). In contrast, the olive leaf extracts showed a noticeable improvement in all these biomarkers (*p* < 0.01) with a greater effect on the hydroxytyrosol-rich extract.

### 3.6. Liver Oxidative Stress Parameters

**Figure 2:**
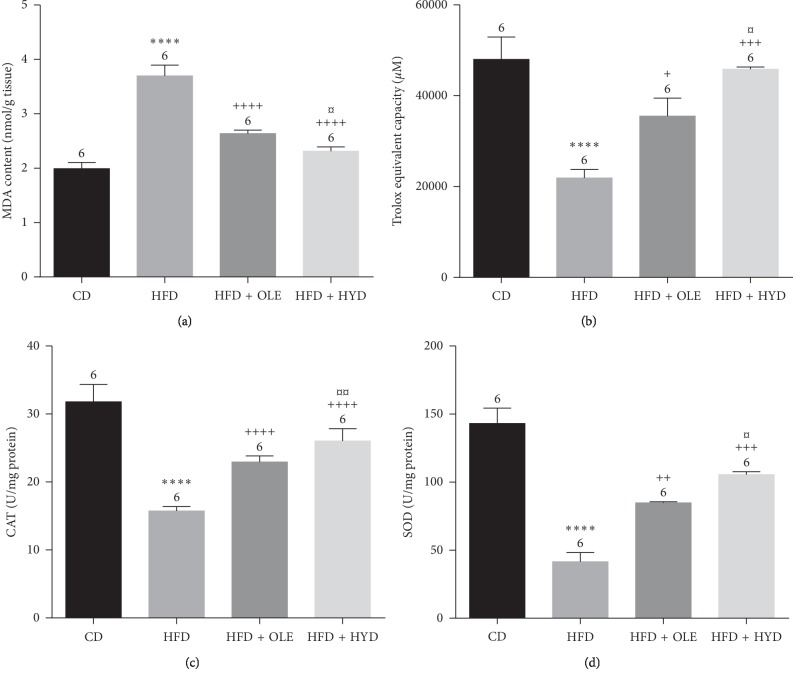
Effect of HFD and oleuropein- and hydroxytyrosol-rich olive leaf extracts on total antioxidant capacity, lipid peroxidation, and on antioxidant status: catalase (CAT) and superoxide dismutase (SOD). Control group (CD), treated group with HFD (HFD), treated group with HFD and oleuropein (HFD + OLE), and treated group with HFD and hydroxytyrosol (HFD + HYD). Treated (HFD) vs. Control (CD): ^*∗∗∗∗*^*p* ≤ 0.0001, ^*∗∗∗*^*p* ≤ 0.001, ^*∗∗*^*p* ≤ 0.01, ^*∗*^*p* ≤ 0.05. Treated (HFD + OLE, HFD + HYD) vs. Treated (HFD): ^++++^*p* ≤ 0.0001, ^+++^*p* ≤ 0.001, ^++^*p* ≤ 0.01, ^+^*p* ≤ 0.05. Treated HFD + OLE vs. Treated HFD + HYD: ^¤¤¤¤^*p* ≤ 0.0001, ^¤¤¤^*p* ≤ 0.001, ^¤¤^*p* ≤ 0.01, ^¤^*p* ≤ 0.05. (a) MDA. (b) ABTS. (c) Catalase. (d) SOD.

The oxidative stress was evaluated in the liver by measuring the TBARS, CAT, and SOD levels. As it is shown in Figure 2, a high-fat diet treatment resulted in a remarkable increase of the MDA level (*p* < 0.001) associated with a significant decrease of the CAT and the SOD activities (*p* < 0.0001). The intake of the oleuropein- and hydroxytyrosol-rich extracts in the HFD-fed rats improved significantly (*p* < 0.05) the antioxidant capacity and reduced the lipid peroxidation of liver tissue towards the control values (Figure 2). When compared to the oleuropein extract, the hydroxytyrosol one offers a greater improvement of hepatic antioxidative status in HFD rats.

### 3.7. Plasma Levels of Leptin and TNF-*α*

**Figure 3:**
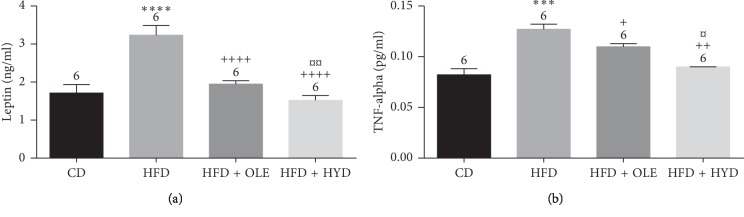
Effect of HFD and oleuropein- and hydroxytyrosol-rich olive leaf extracts on the plasma leptin and TNF-*α* levels: control group (CD), treated group with HFD (HFD), treated group with HFD and oleuropein (HFD + OLE), and treated group with HFD and hydroxytyrosol (HFD + HYD). Treated (HFD) vs. Control (CD): ^*∗∗∗∗*^*p* ≤ 0.0001, ^*∗∗∗*^*p* ≤ 0.001, ^*∗∗*^*p* ≤ 0.01, ^*∗*^*p* ≤ 0.05. Treated (HFD + OLE, HFD + HYD) vs. Treated (HFD): ^++++^*p* ≤ 0.0001, ^+++^*p* ≤ 0.001, ^++^*p* ≤ 0.01, ^+^*p* ≤ 0.05. Treated HFD + OLE vs. Treated HFD + HYD: ^¤¤¤¤^*p* ≤ 0.0001, ^¤¤¤^*p* ≤ 0.001, ^¤¤^*p* ≤ 0.01, ^¤^*p* ≤ 0.05. (a) Leptin. (b) TNF-*α*.

Figure 3 illustrated a significant increase in the plasma leptin and the TNF- *α* levels (*p* < 0.01) in the HFD treated group. The oleuropein and markedly the hydroxytyrosol supplementation at 16 mg/kg showed a significant depletion in all these markers plasma levels (*p* < 0.01).

### 3.8. Histopathological and Immunohistochemical Study of Liver

**Figure 4:**
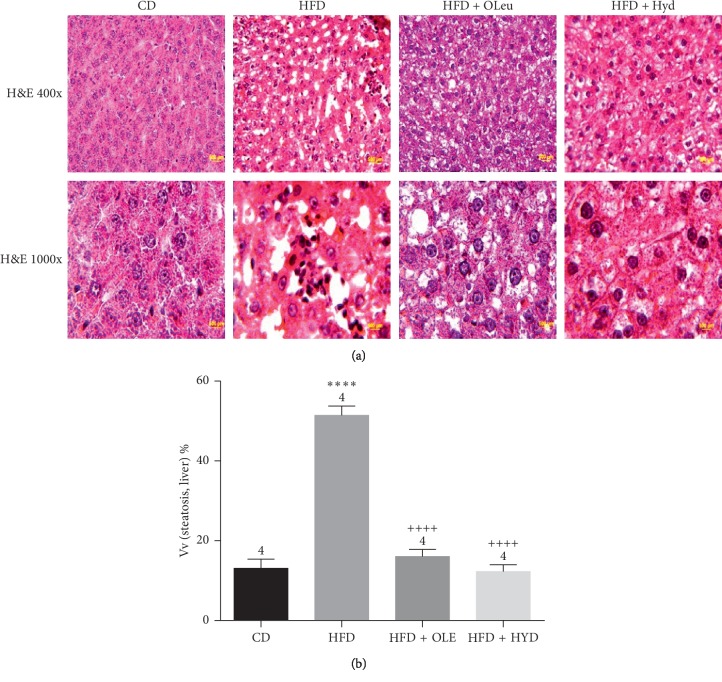
Histological aspect (a) and volume densities (Vv) of steatosis (b) of livers of different groups. Control group (CD), treated group with HFD (HFD), treated group with HFD and oleuropein (HFD + OLE), and treated group with HFD and hydroxytyrosol (HFD + HYD).

**Figure 5:**
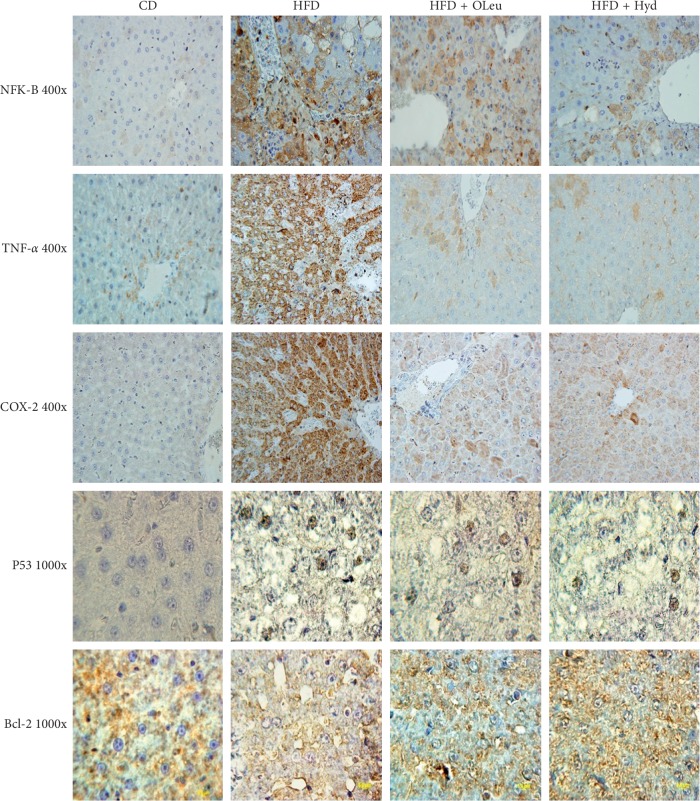
Immunohistochemical staining with anti- TNF-*α*, NF-*κ*b, P53, Bcl-2, and COX-2 in the liver tissues of HFD treated rats (400x). Control group (CD), treated group with HFD (HFD), treated group with HFD and oleuropein (HFD + OLE), and treated group with HFD and hydroxytyrosol (HFD + HYD).

The histopathological analysis of the HFD-fed rats hepatic tissue showed a steatosis and inflammatory damage. Indeed, the hematoxylin-eosin stained sections revealed initial signs of liver inflammation by the existence of inflammatory cell infiltration and a steatosis illustrated by the lipid droplet accumulation in the cytoplasm of the hepatocytes (Figure 4 and Table 7). However, the oleuropein- and hydroxytyrosol-rich olive leaf extracts administration decreased the lipid accumulation and the inflammation in liver tissue, which is clearly displayed by an apparent depletion of nucleus death and inflammatory cells infiltration (Figure 4 and Table 7). In addition, the immunohistochemical study displayed a raise in the P53, COX-2, TNF-*α*, and the NF-*κ*B expression with a decrease in the Bcl-2 expression in the HFD-fed group (Figure 5 and Table 8) with reference to the control group. The administration of the olive leaf extracts decreased the expression of the P53, COX-2 and TNF-*α* considerably. It also improved the Bcl-2 protein expression (*p* < 0.001) (Figure 5 and Table 8).

### 3.9. Histological Study of Adipose Tissue

**Figure 6:**
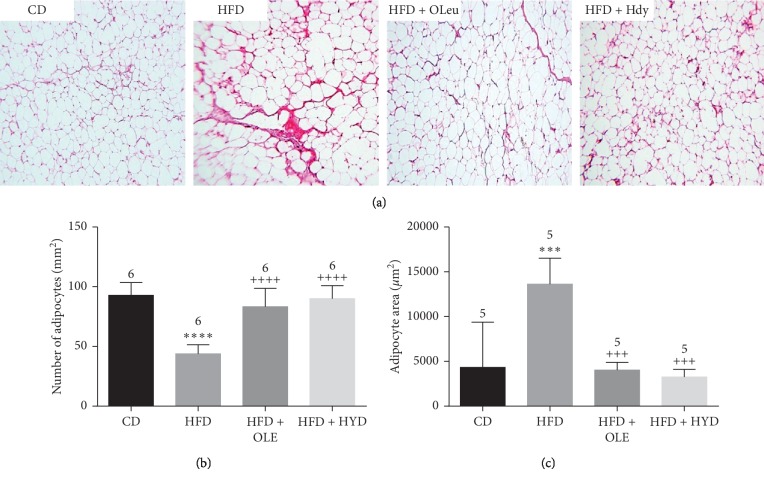
microscopic examination of adipose tissue. Stereological analysis of adipocyte surface area (*μ*m^2^) and number from control and treated rats. Control group (CD), treated group with HFD (HFD), treated group with HFD and oleuropein (HFD + OLE), and treated group with HFD and hydroxytyrosol (HFD + HYD). Treated (HFD) vs. Control (CD): ^*∗∗∗∗*^*p* ≤ 0.0001, ^*∗∗∗*^*p* ≤ 0.001, ^*∗∗*^*p* ≤ 0.01, ^*∗*^*p* ≤ 0.05. Treated (HFD + OLE, HFD + HYD) vs. Treated (HFD): ^++++^*p* ≤ 0.0001, ^+++^*p* ≤ 0.001, ^++^*p* ≤ 0.01, ^+^*p* ≤ 0.05. Treated HFD + OLE vs. Treated HFD + HYD: ^¤¤¤¤^*p* ≤ 0.0001, ^¤¤¤^*p* ≤ 0.001, ^¤¤^*p* ≤ 0.01, ^¤^*p* ≤ 0.05.

The histological study of the adipose tissue in the HFD-fed rats showed hypertrophy of the majority of the adipocytes with membrane rupture. Indeed, an increase in the adipocyte surface area with a decrease in adipocyte number as compared to the control groups (*p* < 0.001), were detected in HFD-fed rats (Figure 6). The supplementation of the oleuropein- and hydroxytyrosol-rich olive leaf extracts (Figure 6) revealed obvious protection of the adipose tissue aspect, which is confirmed by a decrease in the surface area of the majority of the adipocyte and an increase in these numbers with reference to the HFD-fed rats (*p* < 0.001).

## 4. Discussion

Researches of the new natural compounds addressing the management of obesity increase due to their low side effects compared with the conventional pharmacological agents. In this study, the comparative effect of the oleuropein- and hydroxytyrosol-rich extracts derived from olive leaves to modulate lipid metabolism disturbance and liver injury in rats fed with a high-fat diet was reported.

The present data showed that the administration of the oleuropein- and hydroxytyrosol-rich olive leaf extracts identically reduced the body weight, the weight gain, and the epididymal fat accumulation. The biologic events leading to obesity are characterized by excessive growth of the adipose tissue resulting from an increase in the number and/or size of adipocytes [28]. The current histological study of the epididymal adipose tissue showed that the oleuropein- and hydroxytyrosol-rich extracts supplemented rats displayed a significant reduction in the adipocyte size in comparison to the HFD-fed rats. This result implies that the reduction of the body weight after the administration of the olive leaf phenolics is confirmed by an improvement in the histological feature of the HFD-induced adipocyte hypertrophy. Since the adipocyte size was reduced and the cumulative energy intake did not differ between the HFD and HFD + OLE or HFD + HYD groups, it is highly likely to hypothesize that the fat-pad weight lowering effect of oleuropein and hydroxytyrosol could be mediated by the inhibition of the adipogenesis [29, 30].

Moreover, along with the body weight and the adipose mass gains, the HFD-fed rats in the present study displayed increased plasma leptin, which enhanced the fat mass and promoted the cholesterol ester synthesis in the macrophages in a hyperglycemic environment [31]. The increase of the leptin concentration may function as an acute proinflammatory response to prevent excessive cellular stress, which was confirmed by the augmented TNF-*α* level in the HFD-fed rats. In this investigation, it was found that the oleuropein- and hydroxytyrosol-rich olive leaf extracts supplement reduced the serum leptin significant levels in line with the inflammation markers. It was a suggestion of the beneficial effects of the olive leaf phenolics against the inflammation associated with obesity.

When compared to the oleuropein extract, the hydroxytyrosol one offers greater improvement of the leptin and the TNF-*α* levels.

Obesity is well-known by its accompaniment with the alteration in blood lipid profiles such as elevated TG, TC, LDL-C levels which lead directly to liver damage [32]. The present results revealed high levels of TG, TC, and LDL-C and low HDL-C level in the HFD treated rats particularly if it is compared to the control group. The supplementation of the HFD rats with the oleuropein- and hydroxytyrosol-rich olive leaf extracts improved the circulating parameters related to dyslipidemia. However, the hydroxytyrosol treatment is more beneficial for reducing the LDL-C level and increasing the HDL-C level than the oleuropein administration. This result suggested that the olive leaf phenolic compounds extracts exert their hypolipidemic effect on the HFD treated rats by modeling the lipid metabolism. These findings are consistent with the earlier studies indicating the hypolipidemic effect of the olive leaf phenolics in high-fat and high-cholesterol diet rats [16, 30].

The association of obesity with type 2 diabetes has been known for decades. The ability of obesity to produce insulin resistance represents the major basis for this link [33]. Insulin resistance does not only play an important pathophysiological role in the development of type 2 diabetes, but it also takes part as a common component of the metabolic abnormalities, which leads to coronary heart disease, hyperlipidemia, and hypertension [34]. Our study demonstrated that the oleuropein and the hydroxytyrosol-rich extract supplementation lowered the serum insulin activity markedly and improved the insulin sensitivity in the HFD-fed rats. When it is compared to the oleuropein extract, it was found that the hydroxytyrosol extract was more effective in the restoration of the glucose, insulin, and the HOMA-IR amounts. These results were consistent with the previous report of Wang et al. [35] who showed that the olive phenolics had the potential to improve the HFD-induced insulin resistance in mice.

Nonalcoholic fatty liver diseases (NAFLD) constitute the main common cause of chronic liver disease provoked by obesity [36]. The main characteristics of NAFLD are the abnormal fat accumulation in hepatocytes and the inflammatory evolution to steatohepatitis [37]. In this study, the consumption of the HFD caused a significant increase in the relative weight of the liver and the serum AST and ALT levels. It was suggested that the hepatic triglycerides accumulation has occurred in response to the augmented influx of the fatty acids from the adipose tissue to the liver and from the improved endogenous fatty acids synthesis [38]. Indeed, the microscopic examination of HFD rats liver section revealed vacuolated hepatocytes, inflammatory cellular infiltration, pyknotic nuclei, and apoptosis. Previous studies reported that the HFD promotes lipid accumulation in hepatocytes and causes cell infiltration and necrosis [39]. Several reports demonstrated that the elevated liver enzymes AST and ALT indicate the cellular leakage and the loss of the functional integrity of the hepatocyte membrane architecture. These two enzymes are considered as suitable markers for liver inflammation and necrosis. The current results disclosed that the administration of the oleuropein- and hydroxytyrosol-rich olive leaf extracts ameliorated efficiently the deleterious effects of the HFD on the histopathological and the biochemical events as evidenced by a significant reduction in the relative liver weight and the activities of AST, ALT, and LDH. However, the hydroxytyrosol supplementation is more beneficial for the recovery of the liver function than oleuropein treatment.

The oxidative stress plays a pivotal role in the development of the NAFLD, particularly in the progression from the steatosis to the steatohepatitis [40]. It is plausible that the high levels of saturated fatty acids and hyperglycemia can encourage a greater activation of the NADPH oxidase, in addition to a lower formation of the antioxidant enzymes. Accordingly, they led to an imbalance between the reactive oxygen species formation and the antioxidant protection featuring oxidative stress [41]. Mitochondria constantly exposed to the high levels of the reactive oxygen species (ROS) can suffer from structural and functional alterations. They may contribute to the reduction of the beta-oxidation leading to a greater accumulation of free fatty acid [42]. In the present study, the exposure to the HFD-induced an increase of the lipid peroxidation and conversely a decrease in the total antioxidant capacity. The latter is associated with a decrease in the activity of the antioxidant liver enzyme activity such as CAT and SOD. The oleuropein- and hydroxytyrosol-rich olive leaf extracts could diminish the oxidative stress through lowering the MDA levels and elevating the antioxidant enzyme activities in the HFD-fed rats. However, the hydroxytyrosol extract offers greater improvement of the hepatic antioxidative status in the HFD rats, in case it is compared to the oleuropein extract.

These finding are in an agreement with the study of Pirozzi et al. [43] who demonstrated that the hepatoprotective effects of hydroxytyrosol in the HFD animal model were mediated through the improvement of the mitochondrial function as well as the decrease of the oxidative stress via the activation of the AMPK pathway.

Pathological increases in oxidative stress linked with the apoptosis of the hepatic cells have emerged as an important mechanism applied with the development and the progression of the NAFLD. The apoptosis of the hepatocyte is a characteristic of the NAFLD. It has been reported in both patients and experimental animals [44]. It counts as one of the possible mechanisms of the oxidative stress inducing the hepatocyte transition from fatty liver to steatohepatitis. Targeting the hepatocyte apoptosis could be efficient in modulating the NAFLD [45]. In this investigation, the immunohistochemical data of the liver damage induced by the HFD showed an increase in the expression of the P53 coupled with a decrease in the expression of the Bcl-2. This finding is in agreement with the report of Jiang et al. [46] who showed the raise of the expression of caspase-3 and cytochrome c in the hepatic cells of rats fed with high-fat diet for a range from 12 to 16 weeks. The administration of oleuropein and hydroxytyrosol protected the liver tissue from apoptosis mainly by the decrease in the expression of the P53 coupled with an increase in the expression of the Bcl-2. Similar to the current results are several studies which have reported the antiapoptotic effects of the olive leaf phenolics. In this respect, the oleuropein- and hydroxytyrosol-rich olive leaf extracts have been demonstrated to attenuate the liver apoptosis and the lipid metabolism disturbance in the HFD-fed rats [47]. In addition, the preventive effect of hydroxytyrosol against the HFD-induced liver damage and mitochondrial dysfunction in the obese mice has been revealed [48]. Furthermore, Alhaithloul et al. [49] have illustrated that olive leaf extract inhibited the cyclophosphamide-induced oxidative stress and the apoptosis in rat kidney.

Inflammation is also known as an important mediator in the progression from the NAFLD to the NASH. The hepatic inflammatory response is mediated by the proinflammatory cytokines, particularly the TNF-*α*, which is one of the first adipokines released in many types of liver injury. It modulates the effects of the other cytokines. Previous data have illustrated a significant correlation between the HFD and the inflammatory markers such as the IL-6 and the TNF-*α*. Moreover, the nuclear factor kappa B (NF-*κ*B) plays a major role in regulating the immune response to the inflammatory mediators. In the immunohistochemical study, it was shown that the treatment with the HFD increases the expression of the COX-2, NF-*κ*B, and TNF-*α* significantly in relation to the untreated control. Our findings are in agreement with the study of Roh et al. who demonstrated an increase of the mRNA levels of the TNF-*α*, CD_68_, and MCP-1 in the liver of the HFD mice [50]. Equally, it was shown that oleuropein and hydroxytyrosol repressed the liver inflammation and downregulated the expression of the COX-2, TNF-*α*, and NF-*κ*B. This result went with those of Potocnjak and collaborators [51]. They showed that the oral administration of oleuropein diminished the expression of the NF-*κ*B, TNF-*α*, and COX-2 in the kidneys upon the cisplatin treatment. In the same case, Silva et al. [52] have demonstrated that the hydroxytyrosol supplemented refined olive oil showed anti-inflammatory mechanism which inhibited the iNOS and the COX-2 expression, in the animal models of the acute inflammation and the rheumatoid arthritis.

## 5. Conclusion

This study was an evaluation of the protective effect of the oleuropein- and hydroxytyrosol-rich olive leaf extracts on high-fat diet-induced lipid metabolism disturbance and liver injury in rats. Both extracts, mainly the hydroxytyrosol-rich extract, were strongly effective in the protection against body weight gain by preventing the white adipose tissue overgrowth. In addition, these extracts were capable of protection against the lipid metabolism perturbation and the degenerative changes in the hepatic cells induced by the HFD, not only by enhancing the antioxidant system activity in the cells but also by blocking the expression of the proteins involved in the inflammation and liver damage.

## Figures and Tables

**Table 1:** Composition of the experimental diets.

Ingredients (g/kg)	Normal diet	High-fat diet
Moisture	140	112
Proteins	220	176
Fat	35	28
Sheep fat	—	170
Corn oil	—	30
Ash	67	53.6
Fibers	34	27
Carbohydrates	465	372
Mineral mix	13.5	10
Vitamin and antioxidant	4.5	3.6
Amino acid	21	16.8
Bile salts	—	1

**Table 2:** Phenolic compounds detected in olive leaf extract (OLE) with their HPLC retention times.

Compounds	Retention time (min)	Content (mg/g of OLE)
Oleuropein	18.91	905.96
Hydroxytyrosol	10.10	2.28
Verbascoside	15.43	5.36
Luteolin-7-glucoside	15.84	8.78
Apigenin-7-glucoside	17.57	12.06

**Table 3:** Phenolic compounds detected in olive leaf hydrolysate extract (OLHE) with their HPLC retention times.

Compounds	Retention time (min)	Content (mg/g of OLHE)
Hydroxytyrosol	9.917	53.29
Tyrosol	12.809	0.71
Verbascoside	15.503	0.62
Luteolin-7-O-glucoside	15.752	0.79
Apigenin-7-O-glucoside	17.395	4.93
Oleuropein	18.975	0.66

**Table 4:** Effect of HFD and oleuropein- and hydroxytyrosol-rich olive leaf extracts on body growth parameters of rats. Control group (CD), treated group with HFD (HFD), treated group with HFD and oleuropein (HFD+OLE) and treated group with HFD and hydroxytyrosol (HFD + HYD).

Parameters	CD	HFD	HFD + OLE	HFD + HYD
Initial body weight (g) (8)^a^	235 ± 5	235.7 ± 5	246.25 ± 5	248.62 ± 5
Final body weight (g) (8)	287 ± 4	360 ± 7^*∗∗∗∗*^	292 ± 2^++++^	294 ± 5^++++^
Body weight gain (g) (8)	52 ± 2	124.7 ± 3^*∗∗∗∗*^	45.75 ± 10^++++^	45.38 ± 8^++++^
Body mass index (g/cm^2^) (8)	0.16 ± 0.01	0.24 ± 0.05^*∗∗∗∗*^	0.2 ± 0.01^++++^	0.2 ± 0.02^++++^
Relative weight of epididymal fat (mg/g BW.) (8)	4.47 ± 0.8	9.28 ± 0.9^*∗∗∗∗*^	6.34 ± 0.5^++++^	5.20 ± 0.6^++++¤^

^a^Number of determinations. Treated (HFD) vs. Control (CD): ^*∗∗∗∗*^*p* ≤ 0.0001, ^*∗∗∗*^*p* ≤ 0.001, ^*∗∗*^*p* ≤ 0.01, ^*∗*^*p* ≤ 0.05. Treated (HFD + OLE, HFD + HYD) vs. Treated (HFD): ^++++^*p* ≤ 0.0001, ^+++^*p* ≤ 0.001, ^++^*p* ≤ 0.01, ^+^*p* ≤ 0.05. Treated HFD + OLE vs. Treated HFD + HYD: ^¤¤¤¤^*p* ≤ 0.0001, ^¤¤¤^*p* ≤ 0.001, ^¤¤^*p* ≤ 0.01, ^¤^*p* ≤ 0.05.

**Table 5:** Effect of HFD and oleuropein- and hydroxytyrosol-rich olive leaf extracts on lipid parameters. Control group (CD), treated group with HFD (HFD), treated group with HFD and oleuropein (HFD + OLE), and treated group with HFD and hydroxytyrosol (HFD + HYD).

Parameters	CD	HFD	HFD + OLE	HFD + HYD
TC (8)^a^	1.07 ± 0.01	1.64 ± 0.03^*∗∗∗∗*^	1.22 ± 0.04^++^	1.16 ± 0.02^++++¤^
HDL (8)	0.5 ± 0.05	0.22 ± 0.01^*∗∗∗∗*^	0.34 ± 0.05^++^	0.45 ± 0.06^++++¤¤^
LDL (8)	0.39 ± 0.05	0.96 ± 0.07^*∗∗∗∗*^	0.725 ± 0.03^++^	0.44 ± 0.04^++++¤¤^
TG (8)	0.83 ± 0.03	2.24 ± 0.05^*∗∗∗∗*^	1.28 ± 0.07^++++^	1.21 ± 0.09^++++¤^

^a^Number of determinations. Treated (HFD) vs. control (CD): ^*∗∗∗∗*^*p* ≤ 0.0001, ^*∗∗∗*^*p* ≤ 0.001, ^*∗∗*^*p* ≤ 0.01, ^*∗*^*p* ≤ 0.05. Treated (HFD + OLE, HFD + HYD) vs. treated (HFD): ^++++^*p* ≤ 0.0001, ^+++^*p* ≤ 0.001, ^++^*p* ≤ 0.01, ^+^*p* ≤ 0.05. Treated HFD + OLE vs. treated HFD + HYD: ^¤¤¤¤^*p* ≤ 0.0001, ^¤¤¤^*p* ≤ 0.001, ^¤¤^*p* ≤ 0.01, ^¤^*p* ≤ 0.05.

**Table 6:** Effect of HFD and oleuropein- and hydroxytyrosol-rich olive leaf extracts. Liver parameters changes: relative liver weight (mg/g B.W.); plasma levels of ALT (UI/L), AST (UI/L), PAL (UI/L), and LDH (UI/L). Control group (CD), treated group with HFD (HFD), treated group with HFD and oleuropein (HFD + OLE), and treated group with HFD and hydroxytyrosol (HFD + HYD).

Parameters	CD	HFD	HFD + OLE	HFD + HYD
Relative liver weight (mg/g B.W) (8)	35.74 ± 1.5	43.09 ± 2^*∗∗∗∗*^	35.19 ± 1.7^++++^	35.53 ± 2.1^++++^
AST (UI/L) (8)^a^	76 ± 3	165.66 ± 5^*∗∗∗*^	100.5 ± 4^++^	98.66 ± 2^++^
ALT (UI/L) (8)	28 ± 1.5	61 ± 2.2^*∗∗∗*^	37.33 ± 1.6^++^	26.5 ± 1^+++¤^
LDH (UI/L) (8)	202.75 ± 5	647.87 ± 3.2^*∗∗∗∗*^	333.5 ± 3.5^+++^	270.5 ± 2.1^++++¤¤^

^a^Number of determinations. Treated (HFD) vs. Control (CD): ^*∗∗∗∗*^*p* ≤ 0.0001, ^*∗∗∗*^*p* ≤ 0.001, ^*∗∗*^*p* ≤ 0.01, ^*∗*^*p* ≤ 0.05. Treated (HFD + OLE, HFD + HYD) vs. Treated (HFD): ^++++^*p* ≤ 0.0001, ^+++^*p* ≤ 0.001, ^++^*p* ≤ 0.01, ^+^*p* ≤ 0.05. Treated HFD + OLE vs. Treated HFD + HYD: ^¤¤¤¤^*p* ≤ 0.0001, ^¤¤¤^*p* ≤ 0.001, ^¤¤^*p* ≤ 0.01, ^¤^*p* ≤ 0.05.

**Table 7:** Incidence of histopathological lesions in the liver tissue of the studied groups. Injury scores: None (0), Mild (1), Moderate (2), and Severe (3). Control group (CD), treated group with HFD (HFD), treated group with HFD and oleuropein (HFD + OLE), and treated group with HFD and hydroxytyrosol (HFD + HYD).

Liver lesions	CD	HFD	HFD + OL	HFD + Hyd
Portal inflammation	0	2	1	1
Degeneration of hepatocytes	0	2	1	1
*Vascular congestion*	0	1	0	0

**Table 8:** Scoring criteria of immunohistochemistry (IHC) assay with specific antibodies used in this study. Control group (CD), treated group with HFD (HFD), treated group with HFD and oleuropein (HFD + OLE), and treated group with HFD and hydroxytyrosol (HFD + HYD).

Staining positive cells			Staining intensity		Final score product	
Groups	Percent%	Score 1	Intensity	Score 2	Score 1 × score 2	Score 3
*NFK-B*						
CD	<5	0	Absent	0	0-1	0(−)
HFD	51–75	3	Strong	3	9–12	3+(+++)
HFD + OL	6–25	1	Weak	1	2–4	1+(+)
HFD + Hyd	6–25	1	Weak	1	2–4	1+(+)

*TNF-α*						
CD	<5	0	Absent	0	0-1	0(−)
HFD	51–75	3	Strong	3	9–12	3+(+++)
HFD + OL	6–25	1	Weak	1	2–4	1+(+)
HFD + Hyd	6–25	1	Weak	1	2–4	1+(+)

*P53*						
CD	<5	0	Absent	0	0-1	0(−)
HFD	51–75	3	Strong	3	9–12	3+(+++)
HFD + OL	26–50	2	Moderate	2	5–8	2+(++)
HFD + Hyd	26–50	2	Moderate	2	5–8	2+(++)

*Bcl-2*						
CD	51–75	3	Strong	3	9–12	3+(+++)
HFD	6–25	1	Weak	1	2–4	1+(+)
HFD + OL	26–50	2	Moderate	2	5–8	2+(++)
HFD + Hyd	26–50	2	Moderate	2	5–8	2+(++)

*COX-2*						
CD	<5	0	Absent	0	0-1	0(−)
HFD	51–75	3	Strong	3	9–12	3+(+++)
HFD + OL	6–25	1	Weak	1	2–4	1+(+)
HFD + Hyd	6–25	1	Weak	1	2–4	1+(+)

Note: scoring results are based on screening 12 consecutive microscopic fields. Percent positive cells (score 1) multiply staining intensity (score 2) equals final product score (score 3). Either 0 or (−) depicts negative staining. For example, an individual slide had <5% of staining cells (=0) with a staining intensity of 1 (=weak) which would generate a final product score of 0 × 1 = 0; another slide had 80% of staining cells (=4) with a staining intensity of 3 (=strong) which would give a final product score of 4 × 3 = 12 (3+).

## Data Availability

The data used to support the findings of this study are included within the article.
